# Comparative Efficacy and Tolerability of Treatments for Erythromelalgia: A Systematic Review

**DOI:** 10.3390/medicina61050920

**Published:** 2025-05-19

**Authors:** Abdullah S. Algarni, Reem M. Alharthi, Shaden O. Alqurashi, Ruba M. Alghanmi, Rimaz R. Aldawsari, Maysaa A. Alghamdi, Ramy Samargandi

**Affiliations:** 1 Department of Medicine, College of Medicine, University of Jeddah, Jeddah 23218, Saudi Arabia; 2 Faculty of Medicine, Al-Baha University, Al-Aqiq 65522, Saudi Arabia; 3 Collage of Medicine, Taif University, Taif 21944, Saudi Arabia; 4 College of Medicine, University of Jeddah, Jeddah 23218, Saudi Arabia; 5 Department of Surgery, College of Medicine, University of Jeddah, Jeddah 23218, Saudi Arabia

**Keywords:** erythromelalgia, treatment efficacy, management, tolerability, pain management, systematic review

## Abstract

*Background and Objectives*: Erythromelalgia (EM) is an uncommon condition marked by recurring redness, intense burning sensations, and elevated limb warmth. This syndrome can be significantly debilitating, and finding effective treatment options often proves to be quite difficult. The symptoms can severely impact the quality of life of those affected, resulting in considerable disability. This systematic review aims to compare available medical treatments for EM by evaluating their efficacy and safety. *Materials and Methods*: Following PRISMA guidelines, the search included the PubMed, Medline, and Web of Science databases, using the keywords (“Erythromelalgia” OR “Mitchell’s Disease”) AND (“Erythromelalgia Treatment” OR “Erythromelalgia Management”). *Results*: From the 103 papers extracted through the database search, six articles were considered suitable for the systematic review. The included studies investigated various interventions used for a total of 120 patients, including iloprost (*n* = 8), misoprostol (n = 21), topical amitriptyline-ketamine (*n* = 36), lidocaine (*n* = 27), chemical lumbar sympathectomy (CLS, *n* = 13), and various pharmacological agents (*n* = 11). The outcomes showed significant improvements in areas like pain reduction, cooling scores, and temperature regulation. Iloprost and misoprostol exhibited notable benefits in cooling scores, sympathetic dysfunction, and EM severity compared to placebos. About 75% of the patients reported pain relief with topical amitriptyline-ketamine, while lidocaine reduced nociceptive feelings in a dose-dependent manner. *Conclusions*: Comparing interventions demonstrated consistent clinical benefit with varied tolerability. However, adverse events ranged from mild gastrointestinal symptoms to severe complications such as disability and depression, requiring careful monitoring. Given EM’s diverse symptoms and comorbidities, treatment efficacy varies among individuals. A personalized approach incorporating genetic testing, multidisciplinary care, and long-term monitoring is essential to optimize outcomes. Continued research is vital to advance understanding of EM’s pathophysiology and improve patient care.

## 1. Introduction

Erythromelalgia (EM) is a rare neurovascular disorder marked by discomfort, redness, and increased limb warmth. While the feet are predominantly involved, the hands, legs, and face may also be affected. A retrospective analysis involving 168 patients revealed that 88.1% had foot involvement, 25.6% experienced symptoms in their hands, 13.7% in their legs, and 2.4% in their faces [1]. For many, EM manifests as an episodic disorder, with symptoms that fluctuate in intensity. Heat worsens the condition, while exposure to cold water often brings relief [2]. EM presents in two distinct forms: primary and secondary. Primary EM is typically associated with autosomal dominant mutations in the *SCN9A* gene, which encodes the Nav1.7 voltage-gated sodium channel involved in pain signaling [3,4]. Approximately 5% of patients report a family history of EM, with many exhibiting autosomal dominant inheritance patterns. Clinically, primary EM often begins in childhood or adolescence, tends to be persistent and symmetrical, and may occur in familial clusters [5,6]. However, not all cases of primary EM are linked to SCN9A mutations; reports of affected families without identifiable variants suggest genetic heterogeneity and the possible involvement of other genes [7]. Secondary EM, in contrast, is not associated with known genetic mutations and typically presents later in life. It is often linked to underlying conditions such as myeloproliferative disorders (e.g., essential thrombocythemia, polycythemia vera), autoimmune diseases, infections, tumors, or medication use [8]. Secondary EM tends to be more variable in presentation, may be unilateral or localized, and treatment usually targets the underlying condition. Although the distinction between primary and secondary EM is well described, its implications for treatment decisions remain unclear. For patients with localized symptoms or who are unable to tolerate systemic therapies, topical treatments remain the mainstay of symptom management [8].

The pathophysiology of primary EM has been closely linked to gain-of-function mutations in the *SCN9A* gene, which encodes the Nav1.7 voltage-gated sodium channel. These channels are primarily expressed in nociceptive sensory neurons of the dorsal root ganglia (DRG) and sympathetic ganglia, where they play a key role in pain signaling [3]. Mutations in *SCN9A* lead to increased neuronal excitability by causing the channels to open more readily and remain open longer, thereby lowering the threshold for activation and promoting repetitive firing of nociceptive neurons—mechanisms that underlie the intense burning pain of EM [4,9,10]. Additionally, these mutations may reduce excitability in sympathetic neurons, potentially contributing to the thermoregulatory symptoms commonly observed in EM patients [4,10]. Understanding these molecular mechanisms supports the rationale for exploring targeted therapies that modulate sodium channel function [4].

Several therapeutic options have been explored for EM. 5% lidocaine applied topically is a widely used option for anesthesia. It functions as an aminoamide anesthetic by preventing sodium movement through voltage-gated sodium channels, which helps stabilize sensory nerve fibers and effectively interrupt the pain sensation [11]. The Mayo Clinic has created a compounded gel containing amitriptyline and ketamine as a potential new therapy for refractory EM. Amitriptyline is an older antidepressant that works by blocking the reabsorption of serotonin and norepinephrine while also inhibiting sodium channels [12]. Moreover, antihistamines can be considered for treatment, but their effectiveness in managing EM is generally limited. While EM doesn’t seem directly linked to histamine, some researchers think that a persistent, localized reaction could play a role, which might validate the use of antihistamines. For instance, a case study reported a child with primary EM who experienced moderate improvement with the administration of oral cetirizine hydrochloride [13].

Despite growing interest in EM, a lack of consolidated, evidence-based guidance remains to support clinical decision-making. Few studies have evaluated the available treatment options, and no consensus exists regarding the optimal first-line therapy or the management of patients who are refractory to multiple interventions. Furthermore, although the literature acknowledges the role of SCN9A mutations, the implications of these genetic findings for therapeutic efficacy remain poorly understood. Therefore, a systematic review of current therapeutic options is warranted. This review aims to address these clinical uncertainties by systematically comparing the efficacy and safety of current treatments, identifying gaps in the literature, and proposing directions for future personalized management strategies in EM.

## 2. Materials and Methods

This systematic review was conducted using the guidelines established by the PRISMA framework for systematic reviews and meta-analyses [14]. The review protocol was registered on PROSPERO (Registration ID: CRD420251013555).

### 2.1. Literature Search Strategy

The search encompassed various databases, including PubMed, Medline, Cochrane, Scopus, and Web of Science. The strategy included the key terms (Erythromelalgia OR “Mitchell’s Disease”) AND (“Erythromelalgia Treatment” OR “Erythromelalgia Management” OR “Pain Management” OR “Neuropathic Pain” OR “Vasculopathy” OR “Burning Pain” OR “Redness” OR “Extremity Pain”) AND (“Iloprost” OR “Lidocaine” OR “Misoprostol” OR “Magnesium” OR “Aminophylline” OR “Amlodipine” OR “Beta Blocking Agent” OR “Pregabalin” OR “Escitalopram” OR “Mexiletine” OR “Venlafaxine” OR “Duloxetine” OR “Rupatadine”).

### 2.2. Inclusion and Exclusion Criteria

This systematic review examined studies on the medical treatment of EM that were published from 1985 to the present and that assessed the effectiveness and safety of pharmacologic or procedural interventions. Effectiveness outcomes included improvement in EM-related symptoms such as pain, erythema, and temperature dysregulation. Safety outcomes included the reporting of side effects associated with the intervention. The inclusion criteria required studies to involve human participants diagnosed with EM, to report clinical outcomes related to symptom relief, and to use either randomized controlled trials or observational designs (prospective, retrospective, or cohort studies). The search was restricted to studies published in English. Studies were excluded if they were duplicates, lacked treatment outcome data, involved unrelated patient populations or interventions, focused exclusively on diagnostic methods or genetic analysis without reporting clinical outcomes, or were part of the grey literature, including conference abstracts and clinical trial registries. For studies involving mixed patient populations, data relevant only to EM were considered.

### 2.3. Selection of Articles and Data Extraction

Considering the article title and abstract, two independent authors reviewed and screened the complete text according to the inclusion and exclusion criteria. Discrepancies in extraction were resolved through consensus or consultation with a third reviewer. Moreover, data were transferred manually and independently into an Excel sheet. The extracted data included the study’s characteristics (the title of the study, first author’s last name, year of publication, country of origin, study design, and name of the journal used for publication) and demographics (age, sample size, number of groups, number of patients in each group, symptoms, triggers, and relievers), in addition to the intervention details (intervention, dose, and route of administration), outcome measures, and adverse events.

### 2.4. Quality Assessment

The Cochrane risk-of-bias tool for randomized trials (RoB2) [15] was used to evaluate the studies’ risk of bias (RoB). This tool evaluates the risk of bias in randomized controlled trials (RCTs) across five domains: the randomization process, deviations from intended interventions, missing data, measurement of outcomes, and selection of reported results. Each domain is assessed for low, unclear, or high bias, and each study’s overall risk of bias is determined based on these assessments. The ROBINS-I [16] (Risk of Bias In Non-randomised Studies—of Interventions) quality assessment tool was also used. This tool is designed to evaluate the risk of bias in non-randomized studies of interventions. It assesses seven domains through which bias might be introduced, including confounding, selection of participants, classification of interventions, deviations from intended interventions, missing data, measurement of outcomes, and selection of the reported result. Each domain is assessed for low, unclear, or high bias, and each study’s overall risk of bias is determined based on these assessments.

## 3. Results

### 3.1. PRISMA Diagram

**Figure 1:**
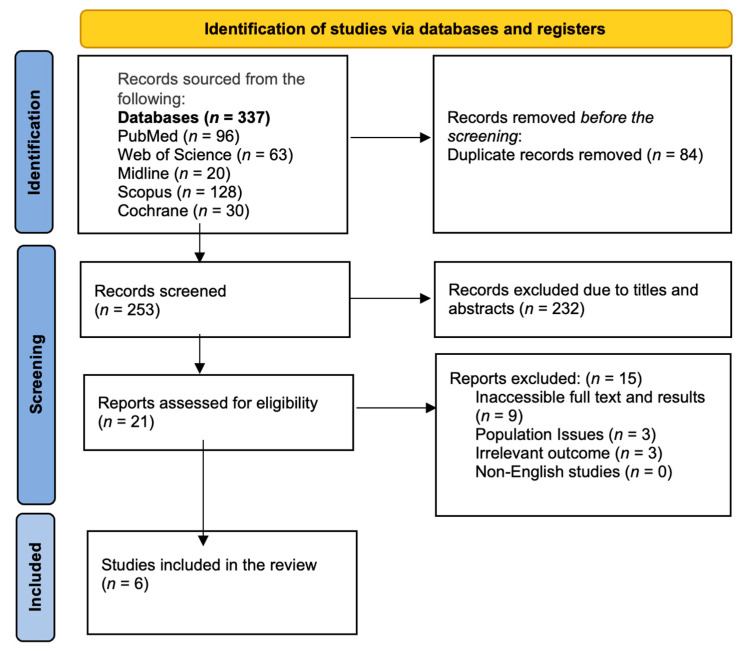
A schematic representation of the criteria used to select studies in the systematic review.

A total of 337 papers were extracted from five databases (PubMed, Medline, Cochrane, Scopus, and Web of Science). Of these, 84 were omitted as duplicates and 232 were excluded due to their titles and abstracts. Six articles were considered suitable for the systematic review (Figure 1).

### 3.2. Overview of Studies Included

A total of 120 patients with EM were included in the review, with ages ranging from 5 to 74 years and disease durations varying from a few months to 34 years. The studies were conducted across different countries, including Norway, Sweden, China, Italy, and the United States, in the period between 2003 and 2023 [17,18,19,20,21,22]. Common comorbidities included small fiber neuropathy, connective tissue disease, myeloproliferative disease, diabetes mellitus, sciatica, and congenital spina bifida. Some patients also had genetic mutations in sodium channel subunits or the *SCN9A* gene. Symptoms typically included burning pain, erythema, and increased skin temperature, often affecting extremities. Triggers for symptoms included warmth, exercise, dependency, tight footwear or gloves, physical activity, immersion, and emotional stress. Common relievers involve cooling methods such as local skin cooling or ice water immersion. Interventions varied and included iloprost infusions (*n* = 8), misoprostol (*n* = 21), topical amitriptyline-ketamine combinations (*n* = 36), lidocaine injections (*n* = 27), chemical lumbar sympathectomy (*n* = 13), and various medications (*n* = 11) such as non-sedating antihistamines, serotonin-norepinephrine reuptake inhibitors, and sodium channel blockers (Table 1).

Table 2 provides an overview of the treatments used for EM and their efficacy. Treatments for EM include iloprost, misoprostol, amitriptyline-ketamine, lidocaine, CLS (chemical lumbar sympathectomy), and other medications. The outcomes show significant improvements in symptoms such as pain reduction, cooling scores, and temperature measures. Prostaglandin analogs like iloprost were effective in reducing cooling scores and sympathetic dysfunction [18]. Misoprostol showed significant improvements in all clinical outcomes but caused severe gastrointestinal toxicity [17]. Topical therapies, such as amitriptyline-ketamine, provided relief for 75% of patients overall; notably, among those with comorbid small fiber neuropathy, 50% experienced improvement compared to only 11% without neuropathy [19]. Lidocaine also demonstrated dose-dependent analgesic effects [20]. CLS offered sustained pain reduction [21], but its invasive nature carries risks, such as thigh pain and the possibility of relapse in patients with SCN9A mutations.

Adverse events ranged from mild reactions like erythema, headache, hypotension [18], and gastrointestinal toxicity [17] to more severe outcomes, including disability and depression [22]. Genetic factors, particularly SCN9A mutations (found in 5–36% of patients), were linked to treatment resistance and relapse. Sodium channel blockers, such as mexiletine, showed partial efficacy in mutation carriers; however, 36% of those patients still remained refractory [22].

Table 3 presents the quality assessment of three RCT studies, evaluated using the ROB-2 quality assessment tool. Two studies had a low bias [18,20] and one had an unclear bias [17].

Table 4 presents the quality assessment of three cohort studies, evaluated using the ROBINS-1 quality assessment tool. One study had a low bias [21], and two had an unclear bias [19,22].

## 4. Discussion

EM is a rare yet impactful condition that significantly affects the lives of those who experience it [1]. The management of EM poses a considerable clinical challenge due to the variability in how patients respond to different treatments and the heterogeneity of the condition itself. This systematic review evaluates the efficacy and tolerability of various treatment options available for EM, including iloprost, misoprostol, amitriptyline-ketamine, lidocaine, and CLS. Each treatment approach has been assessed to provide insights into its potential benefits and limitations when it is used to manage this complex condition.

Although the exact pathogenesis of EM remains partially unclear, recent genetic research has established a strong association between inherited cases and mutations in the *SCN9A* gene, which encodes the Nav1.7 voltage-gated sodium channel [5,9]. This channel, primarily located in sensory and sympathetic ganglia neurons, plays a central role in pain signaling. Gain-of-function mutations in *SCN9A* lower the activation threshold and prolong depolarization, leading to neuronal hyperexcitability and repetitive firing within the DRG, mechanisms strongly implicated in the symptoms of primary EM [9,10,23,24]. Interestingly, Helås et al. [20] found that patients with EM, even those possessing gain-of-function mutations in NaV1.7, showed no changes in their heat pain thresholds [20]. This observation suggests that despite the genetic mutations, there are complexities in how individuals experience pain, hinting at a need for personalized approaches to treatment.

Patients with SCN9A mutations may respond differentially to sodium channel blockers such as lidocaine and mexiletine, which inhibit persistent sodium currents and stabilize neuronal excitability [25,26]. Specific mutations, such as L858F, have been shown to alter the gating properties of Nav1.7 channels, reducing the effectiveness of some blockers while enhancing the response to others. Notably, mexiletine has demonstrated a normalizing effect on these pathological gating changes, which may explain its clinical efficacy in genetically predisposed patients [27,28]. Conversely, individuals without such mutations may experience limited or inconsistent benefits from these therapies, underscoring the value of genetic profiling in guiding personalized EM treatment strategies [3,29]. This approach can improve patient outcomes, offering hope for more effective management of this challenging condition. Given these findings, it is increasingly clear that incorporating thorough genetic testing into the diagnostic process for EM can significantly aid the identification of patients who may respond well to targeted therapies tailored to their genetic profiles. However, current genetic testing technologies cannot always predict phenotypic severity or treatment response. Therefore, while genetic testing holds promise as a tool for personalizing treatment, its clinical utility is still evolving, and results must be interpreted with caution. Future research should focus on better characterizing genetic variants and establishing robust genotype–phenotype correlations to support the development of more precise, mutation-specific therapies.

Kalgaard et al. [18] demonstrated that iloprost substantially decreases cooling scores and improves sympathetic dysfunction, with mild adverse events such as erythema, headache, and hypotension. Mørk et al. [17] showed that misoprostol significantly improves EM severity and temperature measures compared to placebos, although gastrointestinal side effects like diarrhea and nausea are common. Similarly, other findings have shown that iloprost and misoprostol are associated with reduced symptoms and sympathetic dysfunction [30]. Iloprost, a synthetic analog of prostacyclin (PGI₂), binds to IP receptors on vascular smooth muscle cells, leading to vasodilation and inhibition of platelet aggregation. It improves endothelial function and microcirculatory blood flow, which likely explains its impact on cooling scores and sympathetic function in EM patients [18]. Misoprostol, a prostaglandin E1 analog, enhances vasodilation and modulates inflammatory mediators. Its beneficial effects in EM are thought to result from reduced microvascular arteriovenous shunting, thereby improving skin perfusion and thermoregulation [17].

Approximately 75% of patients experienced pain relief with topical amitriptyline-ketamine in the study by Poterucha et al. [19], with minimal adverse events. However, it may influence vascular regulation by blocking serotonin reuptake and impacting sympathetic nerve fibers by inhibiting the reabsorption of noradrenaline. Research on tricyclic antidepressants (such as amitriptyline and imipramine) and SNRIs (like duloxetine) is primarily based on isolated case reports or series, frequently showing inconsistent effects and often involving multiple medications [31].

Wang et al. [21] observed significant pain reduction with CLS, with mild thigh pain as a side effect. Moreover, a case report described a patient with severe PEM in both their feet and lower legs who underwent CLS treatment and was followed for five years. Sequencing of the *SCN9A* gene revealed a polymorphism identified as R1150W. After CLS, the patient experienced a 50% pain reduction, with burning pain, redness, and swelling resolving within four days, and all ulcerations healed in a month. The patient returned to normal exercise five months later, with no relapses in the following five years. Long-term remission was achieved in this PEM case related to the R1150W SCN9A polymorphism [32]. Despite encouraging short-term results, long-term outcomes after CLS remain underreported and variable. The existing studies provide limited data on recurrence, disease progression, and sustained quality of life following the procedure. Relapse may occur, particularly in patients with SCN9A mutations. For example, a study reported that among patients with these mutations who initially responded to CLS, some experienced symptom recurrence within two years post-procedure [21]. Given the invasive nature of CLS and its potential risk of nerve damage, comprehensive longitudinal studies are essential to assess its long-term efficacy, safety profile, and overall role in the management of EM. These observations highlight the critical need for extended follow-up and the identification of reliable predictors of sustained therapeutic benefit.

Lidocaine and mexiletine act as nonselective partial blockers of voltage-gated sodium channels by targeting segment 6 of the alpha subunit’s fourth domain, thereby reducing the duration of action potentials. As an amide-type local anesthetic, lidocaine works by stabilizing nerve membranes and blocking the ion exchanges necessary for generating and transmitting nerve impulses. It has also been explored for use in the treatment of EM as part of clinical trials [25,26]. Helås et al. [20] reported that lidocaine reduced nociceptive feelings in a dose-dependent manner, although no patients exhibited heightened sensitivity to lidocaine. Similarly, in a study involving 34 patients with EM who were treated at a specialized facility, most of those who applied lidocaine 5% patches reported reduced pain. This was in contrast to a group of 10 patients who did not experience any relief when using lidocaine gel [33]. Michelerio et al. [22] emphasized the differing levels of effectiveness among various treatments, identifying antihistamines, venlafaxine, and mexiletine as the most promising options. They also reported the presence of four distinct heterozygous variants of the *SCN9A* gene in four patients. However, they pointed out the occurrence of serious side effects, including suicidal tendencies and restricted mobility [22].

Comparing interventions reveals consistent clinical benefits with varied tolerability. Iloprost and misoprostol improve cooling scores and vascular symptoms; however, misoprostol is associated with gastrointestinal side effects in up to 50% of patients, while iloprost commonly causes headache, erythema, and nausea [17,18]. Topical amitriptyline-ketamine provides partial or complete pain relief in about 75% of cases, with minimal side effects [19]. Lidocaine demonstrates dose-dependent sensory threshold improvements [20], while systemic sodium channel blockers like mexiletine yield varied results depending on genetic background [21,22]. CLS offers sustained benefit in select patients, but requires careful consideration due to procedural risks. These variations emphasize the importance of individualized treatment based on symptom severity, comorbidities, and tolerability.

A multidisciplinary approach involving dermatologists, neurologists, pain specialists, and genetic counselors is essential to provide holistic care and address the diverse aspects of EM. Patient education and support are also critical to help patients and their families cope with the physical and emotional challenges of the condition. Continued research is necessary to explore new therapeutic options, understand the pathophysiology of EM, and identify biomarkers for early diagnosis and treatment response prediction. Long-term monitoring and follow-up are essential to assess treatment efficacy, manage relapses, and adjust interventions as needed, ensuring sustained improvements in patient quality of life.

This review is limited by small sample sizes, which restrict the generalizability of results. Additionally, there is a lack of thorough data on treatment effectiveness, and the short follow-up durations of several studies may fail to capture long-term outcomes. The risk of bias in the included studies further affects the reliability of the findings. While most RCTs showed a low to moderate risk of bias according to the ROB-2 tool, one had an unclear risk of bias due to its limitations in randomization and blinding. Similarly, the cohort studies presented moderate methodological concerns, including potential confounding and incomplete outcome reporting. A key limitation is the substantial heterogeneity across studies. Patient populations varied in terms of EM subtype (primary vs. secondary), genetic background (e.g., SCN9A mutations), and comorbidities. Interventions differed in regard to drug type, dosage, administration route, and treatment duration, and outcome measures, particularly definitions of pain improvement, were inconsistently reported. Some studies relied on visual analog scales, while others used subjective symptom relief or cooling scores. These differences hinder direct comparison, preclude meta-analysis, and limit the generalizability of conclusions. No study performed stratified analysis based on clinical or genetic subtypes, further limiting insights into personalized treatment strategies. Together, these factors may contribute to overestimated efficacy or underreported adverse events. Therefore, the findings should be interpreted with caution, and future studies should incorporate standardized protocols, stratified analyses, and long-term follow-up to enhance comparability and evidence strength.

## 5. Conclusions

EM is a complex condition requiring personalized treatment approaches due to the variability in patient symptoms, comorbidities, and genetic factors. Among the treatments reviewed, topical amitriptyline-ketamine provided the most consistent symptom relief with minimal side effects, while iloprost and misoprostol demonstrated significant vascular benefits but were frequently associated with adverse events such as nausea and gastrointestinal discomfort. Lidocaine showed dose-dependent improvements in sensory thresholds, and mexiletine appeared particularly effective in patients with SCN9A mutations. CLS offered sustained symptom relief in select cases but poses procedural risks and shows variable long-term outcomes. These findings highlight the need for individualized treatment strategies based on symptom severity, genetic profile, and tolerability. A multidisciplinary approach and continuous patient monitoring are essential to optimize clinical outcomes. Future research should prioritize large-scale, multicenter, and longitudinal trials to evaluate long-term safety and efficacy. It should also incorporate genetic and clinical stratification, particularly involving SCN9A variants, to guide personalized therapy, and it should adopt standardized outcome measures across studies to enhance data comparability and evidence synthesis.

## Figures and Tables

**Table 1:** The baseline characteristics of the studies.

First Author, Year	Study Design and Follow-Up	No. of Exp. Patients/ Control	Median (Range) Age (Years)	Gender Male/Female (n)	Median (Range) Duration of Disease (Years)	Comorbidities and Genetic Factors	Symptoms, Triggers, and Relievers	Intervention
Kalgaard et al., 2003 [18]	Double-blind RCT in Norway	12 EM primary bilateral patients [feet] I (iloprost): 8 C (placebo): 4	52 (17–74) I: 39 (17–60) C: 56 (50–74)	4/8 I: 2/6 C: 2/2	9.9 (3.6–23.4) I: 10.5 (5.7–14.9) C: 8.9 (3.6–23.4)	NA	- Symptoms: Pain, erythema, increased skin temperature - Triggers: Warming - Relievers: Cooling	Iloprost IV (3 days) vs. placebo
Mørk et al., 2004 [17]	Double-blind RCT in Norway and Sweden with follow-up for 3 months	21 EM [left toe] patients 11 healthy controls (for physiological evaluation)	EM: 47.8 (21.2–60.6)	EM: 7/14	11.2 (0.1–34.7)	- Comorbidities: Connective tissue disease (*n* = 3), myeloproliferative disease (*n* = 2), diabetes mellitus (*n* = 2), sciatica (*n* = 2)	- Symptoms: Painful, red, hot extremities - Triggers: Warmth, exercise, dependency of extremity, tight shoes and gloves - Relievers: Cooling	Misoprostol (oral, 6 weeks) vs. placebo (crossover)
Poterucha et al., 2013 [19]	Retrospective study in New York	36 EM in different parts of body [35 lower extremities, 22 hands or upper extremities, 6 face, 5 ears, 1 trunk, and 1 neck]	Mean (SD): 44.7 (15.8) Median (range): 47.8 (5–74)	4/32	NA	NA	NA	Amitriptyline-ketamine (topical) with varying concentrations
Helås et al., 2017 [20]	Double-blind RCT in Norway and Sweden	52 EM patients: 27 (primary and secondary bilateral erythomelal [hands and/or feet]) Healthy controls: 25	Patient group: 55 (29–73) Control group: 26.5 (23–48)	Patient group: 3/24 Control group: 11/14	NA	- Comorbidities: Small fibre neuropathy (in some patients) - Genetic factors: Mutations in sodium channel subunits NaV 1.7, 1.8, or 1.9 (in some patients)	- Symptoms: Pain, redness, warmth - Triggers: Warmth, physical activity - Relievers: Cooling	Lidocaine (intradermal) with varying doses vs. placebo
Wang et al., 2018 [21]	Prospective study in China with average six-year follow-up.	13 primary recalcitrant bilateral EM [lower extremities]	15 (11–52)	4/9	36 (2–120) months	- Comorbidities: Congenital spina bifida, popliteal artery entrapment syndrome, hypertension, hypothyroidism - Genetic factors: *SCN9A* gene mutations identified in 5 patients	- Symptoms: Burning sensation, erythema, increased skin temperature - Triggers: Warmth, immersion, overcooling - Relievers: Cooling	Chemical lumbar sympathectomy (5% phenol topical)
Michelerio et al., 2023 [22]	Retrospective study in Italy with average five-year follow-up.	11 primary bilateral EM [limbs]	Mean (range): 36 (16–57)	Females: 11	NA	- Genetic factors: Four different heterozygous variants of *SCN9A* gene among four patients.	- Symptoms: Erythema, edema, and dysesthesias - Triggers: Heat, humidity, emotional stress, sun exposure, alcohol, evening - Relievers: Temperatures, ice water immersion, limb elevation	- Desloratadine and rupatadine (combination therapy) - Magnesium pidolate supplementation - SNRIs: venlafaxine or duloxetine - Mexiletine with varying doses - Other treatments included Cardioaspirin, Amlodipine, Escitalopram, beta-blockers, and Pregabalin

**Table 2:** The outcomes of the studies.

First Author, Year	Outcomes	Adverse Events	Conclusion
Kalgaard et al., 2003 [18]	**Change from pre-treatment:** Iloprost (*n* = 8) Cooling score: improved (*p* < 0.05) Valsalva’s manoeuvre (b): improved (*p* < 0.05) Contralateral cooling test (b): improved (*p* < 0.05) Placebo (*n* = 4) Cooling score (1–8): improved Valsalva’s manoeuvre (b): improved Contralateral cooling test (b): improved	Erythema: 5 Headache: 5 Warm sensation at the injection site: 2 Nausea: 2 Hypotension: 1	Iloprost significantly reduced cooling scores in erythromelalgia patients compared to the baseline, showing improvements in symptoms and sympathetic dysfunction.
Mørk et al., 2004 [17]	**Change from pre-treatment:** EM Severity (Pain VAS, mm) Baseline: worsened (*p* ≤ 0.01) Placebo: worsened (*p* ≤ 0.01) Misoprostol: no change Temperature (°C) Baseline: improved (*p* ≤ 0.01) Placebo: improved (*p* ≤ 0.01) Misoprostol: improved (*p* ≤ 0.01) Control: improved (*p* ≤ 0.01) LDPI-Assessed Skin Perfusion (AUa) Baseline: improved (*p* ≤ 0.01) Placebo: improved (*p* ≤ 0.01) Misoprostol: improved (*p* ≤ 0.01) Control: improved (*p* ≤ 0.01) Capillary Density (AUa) Baseline: worsened (*p* ≤ 0.01) Placebo: worsened (*p* ≤ 0.01) Misoprostol: worsened (*p* ≤ 0.01) Control: no change Mutual Distance (AUa) Baseline: worsened (*p* ≤ 0.01) Placebo: worsened (*p* ≤ 0.01) Misoprostol: worsened (*p* ≤ 0.01) Control: no change	Abdominal pain: 7 Nausea: 3 Loose stool/diarrhea: 22 Vomiting: 2 Flatulence: 4 Sore throat: 2 Headache: 1 **Placebo** Nausea: 1 Loose stool/diarrhea: 6 Flatulence: 1 Sore throat: 5 Insomnia: 1	Significant improvements in all clinical outcomes after treatment with misoprostol compared to a placebo, even after a three-month follow-up.
Poterucha et al., 2013 [19]	Relief Using Amitriptyline-Ketamine: Presence of small fiber neuropathy: 50% improved Involvement of hands or face: 11% improved Efficacy: Completely improved: 3% Significant improvement: 39% Some improvement: 33%	Redness of the face: 1 Worsening Raynaud’s with erythromelalgia.: 1	About 75% of erythromelalgia patients found pain relief with a topical combination of amitriptyline and ketamine, and the treatment was well accepted.
Helås et al., 2017 [20]	**Warmth Detection Threshold (WD °C)** Saline and low lidocaine (≤0.50 mg/mL): no change. 1.0 mg/mL and 10 mg/mL lidocaine: improved in both Control (*p* < 0.001) and EM (*p* ≤ 0.01 to <0.001). **Heat Pain Detection Threshold (HPT °C)** Saline and low lidocaine (≤1.0 mg/mL): no change. 10 mg/mL lidocaine: improved in both groups (*p* < 0.001). **Heat Pain Sensitivity (HPS NRS)** Saline and low lidocaine (≤0.50 mg/mL): no change. 1.0 mg/mL lidocaine: improved in controls (*p* < 0.05) and EM (*p* ≤ 0.01). 10 mg/mL lidocaine: improved in both (*p* < 0.001). **Cold Detection Threshold (CD °C)** Saline: no change. 0.25 mg/mL lidocaine: improved in controls (*p* < 0.05). 0.50–10 mg/mL lidocaine: improved in controls (*p* < 0.001); EM improvement from 1.0 mg/mL onward (*p* ≤ 0.001). **Dynamic Mechanical Sensitivity (DMS Y/N)** Saline and low lidocaine (≤1.0 mg/mL): no change. 10 mg/mL lidocaine: improved in both groups (*p* < 0.001). **Static Mechanical Sensitivity (SMS NRS)** Saline: no change. 0.25–1.0 mg/mL lidocaine: improved in both groups (*p* ≤ 0.01 to <0.05). 10 mg/mL lidocaine: improved in both (*p* < 0.001). **Mechanical Pain Sensitivity (MPS NRS)** Saline: no change. 0.25–1.0 mg/mL lidocaine: improved in both groups (*p* ≤ 0.01 to <0.05). 10 mg/mL lidocaine: improved in both (*p* < 0.001) at all force levels (128–512 mN).	NA	Although lidocaine reduced nociceptive feelings in a dose-dependent manner, no patients showed heightened sensitivity to it, limiting insights for potential treatments.
Wang et al., 2018 [21]	**The VAS value from the baseline:** Day one: decreased One week: further decreased Three months: maintained after CLS treatment	Mild thigh pain: 3	CLS shows promise for refractory erythromelalgia, with notable improvements but possible relapses, especially in mutation carriers.
Michelerio et al., 2023 [22]	Reduction in pain intensity and episodes: 3 patients. Sufficient improvement with therapy: 4 patients. Poor response to multiple treatments: 4 patients.	Suicidal thoughts: 1 Persistent edema and pain leading to severe walking limitations: 1	The most effective therapies were antihistamines, venlafaxine, and mexiletine.

**Table 3:** A ROB-2 quality assessment of eight RCT studies.

Study ID	D1	D2	D3	D4	D5	Overall ROB-2
Kalgaard et al., 2003 [18]	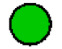	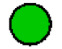	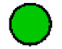	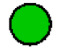	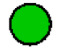	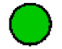
Mørk et al., 2004 [17]	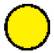	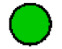	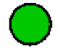	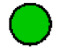	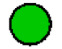	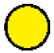
Helås et al., 2017 [20]	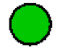	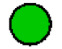	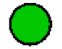	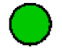	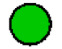	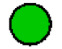

Domains: D1: Bias arising from the randomization process; D2: Bias due to deviation from intended interventions; D3: Bias due to missing data; D4: Bias in measurements of outcomes; D5: Bias in the selection of reported results. Judgment: 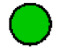: Low bias; 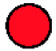: High bias; 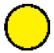: Unclear bias.

**Table 4:** A ROBINS-I quality assessment of cohort studies.

Study ID	D1	D2	D3	D4	D5	D6	D7	Overall RoB
Poterucha et al., 2013 [19]	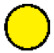	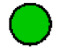	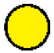	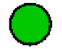	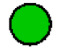	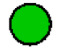	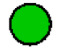	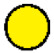
Wang et al., 2018 [21]	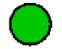	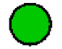	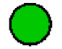	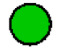	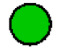	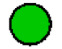	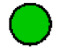	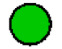
Michelerio et al., 2023 [22]	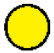	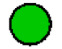	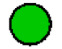	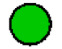	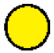	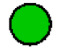	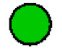	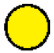

Domains: D1: Bias due to confounding; D2: Bias in the selection of participants for the study; D3: Bias in the classification of interventions; D4: Bias due to deviation from intended interventions; D5: Bias due to missing data; D6: Bias in measurements of outcomes; D7: Bias in the selection of reported results. Judgment: 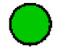: Low bias; 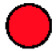: High bias; 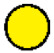: Unclear bias.

## Data Availability

The data presented in this study are available upon request from the corresponding author.

## Abbreviations

The following abbreviations are used in this manuscript:

CLS	Chemical lumbar sympathectomy
EM	Erythromelalgia
RCT	Randomized control trial
ROB	Risk of bias
ROBINS-I	Risk of Bias In Non-randomised Studies—of Interventions
